# Prevalence and risk factors of mental disorders in a cross-sectional study of adolescents in South China

**DOI:** 10.1007/s44192-026-00557-7

**Published:** 2026-07-23

**Authors:** Feifei Wang, Lili Zhang, Xinyu Xiang, Andor Bodnár, Rishi Pednekar, Ruth Cortright, Isabella Overby, Jaspal Mahal, Mengqi Zhang

**Affiliations:** 1 College of Medicine, Jiaxing University, Jiaxing, 314041 China; 2 No. 2 Experimental Primary School, Ninghua County, Sanming City, 365499 Fujian Province China; 3 Department of Health, Human Function, and Rehabilitation Sciences, The George Washington University, Washington, DC 20052 USA; 4 Department of Psychiatry and Behavioral Sciences, Johns Hopkins University School of Medicine, Baltimore, MD 21205 USA; 5 College of Education, Capital Normal University, Beijing, 100048 China

**Keywords:** Student, Mental health, Adolescent, Self-harm, Depression, Anxiety, Stress

## Abstract

**Background:**

The mental health of adolescents is a critical global public health priority. This large-scale, cross-sectional study investigated the prevalence of depression, anxiety, and stress, along with their key associated risk factors, among school-attending adolescents in South China.

**Methods:**

An online anonymous survey utilizing the validated Depression Anxiety Stress Scales-21 (DASS-21) and the Mental Health Test (MHT) was administered to 23,856 students (mean age = 13.57 ± 1.94 years) from primary, middle, and high schools.

**Results:**

The results revealed concerning prevalence rates of mild-to-extreme symptoms: 27.9% for anxiety, 25.15% for depression, and 7.63% for stress. Severe study pressure (reported by 52%), loneliness (59%), and poor social relationships (50%) were identified as the predominant risk factors, with multivariate logistic regression confirming their significant associations with worse mental health outcomes (ORs ranging from 1.74 to 1.95).

**Conclusions:**

This large-scale study quantifies high anxiety and depression prevalence among South Chinese adolescents, identifying study pressure, loneliness, social difficulties, and self-harm as key modifiable risk factors. This study calls for school-based mental health interventions and provides timely empirical evidence to promote student well-being.

## Background

The mental health of adolescents has become a major global concern [1]. Epidemiological data consistently indicate increased rates of depression, anxiety, and stress among youth populations worldwide. According to the World Health Organization (2021), one in seven adolescents experience mental health concerns, with suicide ranking among the leading causes of death for 15- to 19-year-olds. Contributing factors such as academic stress [2], social media use [3], family instability [4–5], and socio-economic inequality [6] have exacerbated the psychological vulnerability of young people. Emerging evidence suggests that the COVID-19 pandemic has further intensified this vulnerability, with lockdowns, school closures, and social isolation contributing to a sustained rise in adolescent internalizing symptoms globally, effects that have extended into the post-pandemic recovery phase [7]. Despite increased awareness, the overall trend suggests a worsening mental health crisis with long-term consequences for education, productivity, and public health.

While national and regional data suggest a surge in adolescent mental disorders, there remains a critical gap in large-scale empirical studies that quantify prevalence and rigorously examine specific, modifiable risk factors. Recent surveys indicate that a significant proportion of Chinese adolescents’ exhibit symptoms of depression and anxiety, and the increasing rate of suicide among adolescents serves as a warning of the serious mental health crisis in this population [8]. Existing studies have reported notable mental health challenges in South China. For example, a study among adolescents in South China found a prevalence of depression of 23.0% and a prevalence of anxiety of 13.9% [9]. Additionally, the rapid urbanization and high population density characteristic of South China may create unique socio-environmental stressors, including intense academic competition and reduced family time due to parental migration for work [10]. However, there is limited research offering robust, large-scale evidence on the specific risk factors contributing to these mental health issues. While academic stress, peer relationships, loneliness, and self-harm behaviors have been extensively documented as risk factors for adolescent mental disorders in quantitative studies [8], large-scale empirical research examining these associations specifically in South China remains limited [10].

Given this context, the present study aims to fill this gap by conducting a large-scale survey across primary, middle, and high schools in South China. Utilizing validated psychological instruments, this research seeks to: (1) quantify the prevalence of depression, anxiety, and stress among adolescents in this region; and (2) empirically examine the association of key psychosocial risk factors (academic pressure, social relationships, loneliness, and self-harm) with these mental health outcomes. The findings are intended to provide timely, evidence-based guidance for targeted school-based interventions and to inform the ongoing evaluation and refinement of national well-being policies.

## Methods

### Study design and participants

This cross-sectional study was conducted in South China using an online survey distributed to primary, middle, and high school students. Included participants were aged 10–19 years, consistent with the World Health Organization’s definition of adolescence. A QR code for the survey, with a study description and consent form, was disseminated to the students via an electronic platform (https://www.wjx.cn/). Data were collected during the beginning of the spring semester (February to April 2024). A total of 31,066 students were invited, of whom 23,856 provided valid data (response rate: 76.79%). The 23.21% non-response rate is acknowledged as a potential source of bias, as students with severe mental distress or limited internet access may have been less likely to participate. Furthermore, the online design inherently excluded students without reliable internet access, which may limit the generalizability of findings to the most socio-economically disadvantaged subgroups. Inclusion criteria were: enrollment in a participating public school, the ability to comprehend the survey questions, and provision of informed consent (with parental consent for minors). Surveys with incomplete responses were excluded.

The questionnaire was distributed in the Chinese language. All students were required to read the consent form prior to completing the survey, by clicking “YES” to continue the survey, and clicking “NO” to close the survey. Students had the right to end the survey at any stage. No financial benefits were provided to the participants. Anonymity was guaranteed to ensure confidentiality and reliability of data. A trained researcher responded to all questions or consultations during the collection period.

### Ethical considerations

The study protocol was approved by the Research Ethics Committee of Jiaxing University (Approval ID: YX-2025008) and the local Education Bureau. The study adhered strictly to the principles of the Declaration of Helsinki. Student assent was also obtained at the start of the online survey, and prior informed consent was obtained from a parent or legal guardian for every participating student. Participation was voluntary, anonymous, and without financial compensation. A critical ethical safeguard was our referral protocol: students whose DASS-21 scores indicated severe levels of depression, anxiety, or stress were automatically flagged. The designated counselor at their respective school was confidentially notified, triggering a standardized procedure to offer follow-up support and referral to local mental health services. Data collection, storage, and analysis complied with the General Data Protection Regulation (GDPR) standards.

## Measurements

Socio-demographic factors: Demographic factors included age, sex, education level, family structure (e.g., two-parent, single-parent), and household urbanicity (urban/rural). Participants were also asked if they had searched for mental health help or received any mental health training courses. Participants were not asked whether they consumed any types of drugs or alcoholic beverages, because those behaviors are prohibited for students under compulsory education.

Mental Health Test (MHT): The Mental Health Diagnostic Test (MHT) was revised from the “Anxiety Tendency Diagnostic Test”, which is applicable to students from primary school through senior high school [11–14]. This survey adopted four contents from eight content scales (i.e., study anxiety, social relations, loneliness, self-harm) considering the length of the survey and attention of students. There were forty questions in the adopted content scale, and each question was answered with “yes” or “no”. Anxiety, social relationship difficulties, loneliness, and self-harm behaviors were calculated by the number of “yes” answers, with higher scores indicating worse mental health status. The standardized Chinese version of the MHT was used without modification. This version has demonstrated good reliability and validity in prior studies with Chinese adolescents [15]. The total MHT score is 100. The scoring ranges for MHT subscales (study anxiety, loneliness, social relations, self-harm) vary from 9 to 15. For each subscale, scores are categorized as follows: severe (> 8), medium (3–8), and not at all (< 3).

Depression, Anxiety and Stress Scale − 21 Items (DASS-21): The DASS-21 is a self-report measure designed to assess the severity of general psychological distress and symptoms related to depression, anxiety, and stress [16–17]. The depression subscale assesses symptoms such as dysphoria, hopelessness, devaluation of life, self-deprecation, lack of interest/involvement, anhedonia, and inertia. The anxiety subscale evaluates symptoms such as autonomic arousal, skeletal muscle effects, situational anxiety, and subjective experience of anxious affect. The stress subscale measures symptoms related to difficulty relaxing, nervous arousal, being easily upset/agitated, being irritable/over-reactive, and impatience. The three subscales of the DASS-21 have been shown to have high internal consistency and to yield meaningful discrimination.

Although the MHT study anxiety subscale and the DASS-21 anxiety scale both assess anxiety-related symptoms, they capture conceptually distinct constructs. The MHT study anxiety is context-specific to academic situations (e.g., exam-related nervousness, fear of teacher questioning), whereas the DASS-21 anxiety measures general, non-academic anxiety symptoms (e.g., autonomic arousal, situational fear, panic experiences). Item-level inspection confirms no overlapping items between the two scales.

### Statistical analysis

Descriptive statistics were used to summarize the socio-demographic factors. Continuous variables were summarized as means and standard deviations (SDs). Categorical variables were summarized as frequencies and percentages. For ordinal variables (e.g., study pressure, loneliness), participants were categorized into three levels: not at all (< 3), medium (3–8), and severe (> 8) based on MHT subscale scores. ANOVA was used for categorical independent variables (e.g., sex, education), which aimed to test whether mean differences in DASS-21 scores across groups were statistically significant. Linear regression was used for the continuous independent variable (e.g., age), which aimed to examine the linear relationship with DASS-21 subscale scores. Multiple logistic regressions were performed to examine the associations of study anxiety, difficulties in social relationships, loneliness, and self-harm behaviors on categorized DASS-21 subscales (depression, anxiety, stress). Each of the three subscales includes 7 items rated on a 4-point Likert scale (0–3). Each subscale score is summed and then multiplied by 2. The categorization of DASS-21 subscales used established cutoff values: depression (0–9: normal, 10–13: mild, 14–20: moderate, 21–27: severe, 28+: extremely severe), anxiety (0–7: normal, 8–9: mild, 10–14: moderate, 15–19: severe, 20+: extremely severe), and stress (0–14: normal, 15–18: mild, 19–25: moderate, 26–33: severe, 34+: extremely severe). The logistic regression models were adjusted for socio-demographic factors such as age, sex, education level, family and urbanicity. The adjusted odds ratios (OR) and 95% confidence intervals (95%CI) were used to estimate associations. Multicollinearity among predictors was examined using variance inflation factors (VIFs), all of which were below 2.5, indicating no significant multicollinearity. Statistical analysis was performed by using SAS (version 9.4, Cary, NC, USA), with significance level set at 0.05.

## Results

### Socio-demographic characteristics

The final analytic sample comprised 23,856 students (Table 1). The mean age of participants was 13.57 years (SD = 1.94; range 10–19 years), with a slight predominance of males (54.7%). Participants were primarily from primary (49.2%) and middle schools (43.0%). A majority lived in two-parent households (68.5%), while a notable proportion lived with a single parent (15.3%) or others (13.9%). Approximately 30% resided in rural areas. Data completeness was high (> 99% for all study variables). For the minimal missing data (< 1%), a complete-case analysis approach was employed, as preliminary analyses confirmed that missingness was not associated with key outcomes. Prior to regression modeling, we examined the correlations among the four main predictors (study pressure, loneliness, social relationship difficulty, self-harm). All pairwise correlations were positive and moderate (*r* range: 0.47–0.56).

**Table 1:** Socio-demographic characteristics of participants (*N* = 23856)

Category	*N* (%)	Depression		Anxiety		Stress	
		Mean(SD)	*p*-value	Mean(SD)	*p*-value	Mean(SD)	*p*-value
Total	23,856(100)	5.58(7.23)	–	5.19(7.18)	–	4.76(6.68)	–
Sex			0.08		< 0.001		< 0.001
Female	10,813(45.3)	5.67(7.11)		5.61(7.29)		5.02(6.75)	
Male	13,043(54.7)	5.51(7.33)		4.85(7.06)		4.54(6.62)	
Age mean (SD)	13.57(1.9)	–	< 0.001	–	< 0.001	–	< 0.001
Education			< 0.001		< 0.001		< 0.001
Primary school	11,726(49.2)	5.06(6.89)		4.59(6.57)		4.12(6.11)	
Middle school	10,266(43.0)	5.89(7.56)		5.59(7.71)		5.17(7.13)	
High school	1864(7.8)	7.18(7.18)		6.77(7.37)		6.52(7.02)	
Family member			< 0.001		< 0.001		< 0.001
Both parents	16,337(68.5)	5.02(6.81)		4.69(6.77)		4.31(6.33)	
Single mother	2989(12.5)	6.42(7.54)		5.93(7.37)		5.43(6.99)	
Single father	677(2.8)	7.21(7.89)		6.48(7.91)		6.02(7.38)	
Orphan	547(2.3)	9.77(10.53)		9.59(10.76)		8.43(9.55)	
Other	3304(13.9)	6.56(7.66)		6.05(7.60)		5.53(7.02)	
Household urbanicity			< 0.001		< 0.001		0.0006
Urban	16,742(70.2)	5.44(6.98)		5.00(6.83)		4.67(6.46)	
Rural	7114(29.8)	5.91(7.77)		5.64(7.91)		4.99(7.17)	

### Prevalence of depression, anxiety and stress

**Fig. 1:**
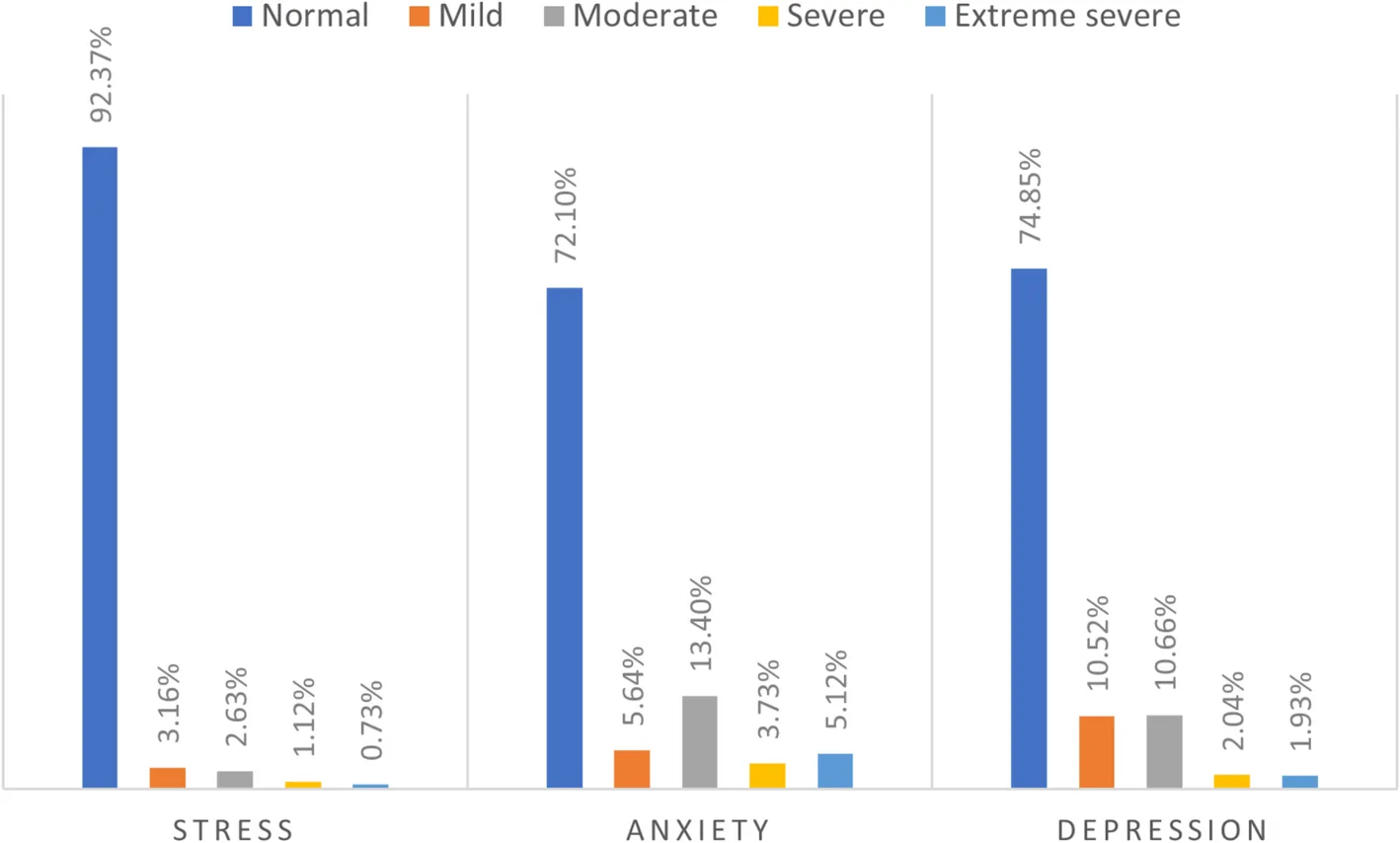
Prevalence of depression, anxiety and stress among adolescents (*N* = 23856)

A larger proportion of students reported mild to extremely severe anxiety (27.9%) and depression (25.2%) than stress (7.6%). Among these participants, 13.40% experienced moderate anxiety, similar to the number of participants who experienced mild (10.5%) or moderate (10.7%) levels of depression. Prevalence of mild-to-extreme symptoms was calculated by summing the proportions of mild, moderate, severe, and extremely severe categories for each outcome as shown in Fig. 1.

**Fig. 2:**
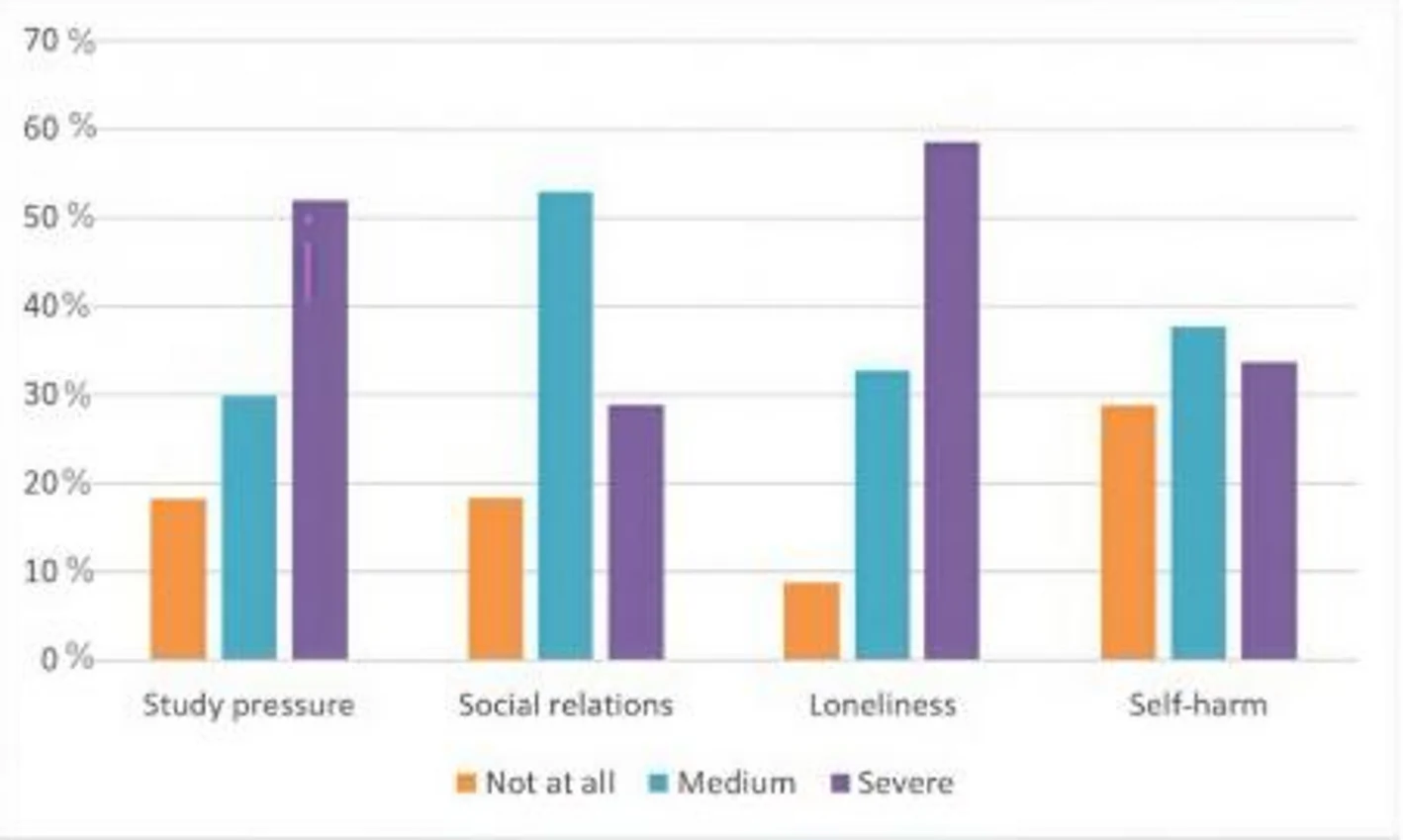
Prevalence of mental disorder related factors among adolescents (*N* = 23856)

Risk factors (e.g., study pressure, social relations, loneliness, self-harm) contribute to mental disorders (Fig. 2). Severe study pressure, loneliness, and moderate level of social relationship difficulty were the dominant risk factors among participants. Loneliness and study pressure (> 50%) are severely prevalent among students in China. The result shows that social relation issue is highly prevalent (50%) at medium level of severity.

### Associations between risk factors and mental health outcomes

Mental health outcomes (i.e., depression, anxiety and stress) showed strong internal consistency among the participants. Table 2 presents associations between risk factors (i.e., study pressure, social relations, loneliness, self-harm) and mental issues (depression, anxiety and stress). The model adjusted for age, sex, education level, family, and urbanicity. According to the multiple logistic regression analysis, increased study pressure, poor social relationships, loneliness, and self-harm were significantly associated with increased levels of depression, anxiety, and stress (*p* < 0.001).

Compared with participants without study pressure, those experiencing moderate study pressure had nearly twice the odds of experiencing symptoms of depression (OR: 1.84; 95% CI: 1.71–1.98, *p* < 0.001). Participants with moderate levels of social relationship difficulty showed even higher odds (OR: 3.82; 95% CI: 3.56–4.09, *p* < 0.001). Similarly, moderate levels of loneliness (OR: 3.31; 95% CI: 3.02–3.62, *p* < 0.001) and self-harm (OR: 2.27; 95% CI: 2.13–2.43, *p* < 0.001) were significantly associated with higher odds of depression. Compared to participants without social relationship problems, those with a moderate level of social difficulties had significantly higher odds of experiencing depression (OR = 3.82; 95% CI: 3.56–4.09, *p* < 0.001), anxiety (OR = 4.06; 95% CI: 3.79–4.35, *p* < 0.001), and stress (OR:4.11; 95%CI:3.70–4.55, *p* < 0.001). Compared to the reference group, participants reporting loneliness had approximately two to three times higher odds of depression (OR = 3.31; 95% CI: 3.02–3.62, *p* < 0.001), anxiety (OR = 2.69; 95% CI: 2.46–2.94, *p* < 0.001), and stress (OR = 2.94; 95% CI: 2.62–3.30, *p* < 0.001). Similarly, those reporting self-harm showed elevated odds for depression (OR = 2.27; 95% CI: 2.13–2.43, *p* < 0.001), anxiety (OR = 2.42; 95% CI: 2.27–2.58, *p* < 0.001), and stress (OR = 2.78; 95% CI: 2.49–3.09, *p* < 0.001).

**Table 2:** Associations between risk factors and depression, anxiety and stress (*N* = 23856)*

Risk factors	Frequency (%)	Depression		Anxiety		Stress	
		OR(95%CI)	*p*-value	OR(95%CI)	*p*-value	OR(95%CI)	*p*-value
*Study pressure*							
Not at all	4336(18.18)	Ref		Ref		Ref	
Medium	7117(29.83)	1.84(1.71–1.98)	< 0.001	1.74(1.61–1.87)	< 0.001	1.95(1.74–2.18)	< 0.001
Severe	12,403(51.99)	5.56(5.14–6.01)	< 0.001	6.27(5.81–6.77)	< 0.001	6.46(5.67–7.36)	< 0.001
*Social relations*							
Not at all	4352(18.24)	Ref		Ref		Ref	
Medium	12,617(52.89)	3.82(3.56–4.09)	< 0.001	4.06(3.79–4.35)	< 0.001	4.11(3.70–4.55)	< 0.001
Severe	6887(28.87)	12.92(11.65–14.32)	< 0.001	17.17(15.47–19.06)	< 0.001	16.20(13.22–19.85)	< 0.001
*Loneliness*							
Not at all	2070(8.68)	Ref		Ref		Ref	
Medium	7820(32.78)	3.31(3.02–3.62)	< 0.001	2.69(2.46–2.94)	< 0.001	2.94(2.62–3.30)	< 0.001
Severe	13,966(58.54)	14.77(13.42–16.26)	< 0.001	13.16(11.98–14.46)	< 0.001	17.06(14.71–19.79)	< 0.001
*Self-harm*							
Not at all	6847(28.70)	Ref		Ref		Ref	
Medium	8977(37.63)	2.27(2.13–2.43)	< 0.001	2.42(2.27–2.58)	< 0.001	2.78(2.49–3.09)	< 0.001
Severe	8032(33.67)	6.26(5.75–6.82)	< 0.001	8.76(8.04–9.56)	< 0.001	10.07(8.44–12.01)	< 0.001

* adjusted age, sex, education level, family, urbanicity

Not at all: <3; Medium:3–8; Severe:>8

## Discussion

This large-scale cross-sectional study provides contemporary empirical evidence on the prevalence of psychological distress and its key psychosocial correlates among adolescents in South China. Our findings indicate that a substantial proportion of students experience clinically significant symptoms, with anxiety (27.9%) and depression (25.2%) being notably more prevalent than stress (7.6%), which align with those reported in previous studies among Chinese adolescents [8]. Importantly, we identified severe study pressure, loneliness, social relationship difficulties, and self-harm as significant factors associated with increased odds of these mental health problems, even after adjusting for key demographic covariates. The rates in our South China sample are also consistent with post-pandemic meta-analyses indicating a global surge in adolescent internalizing symptoms, with pooled prevalence estimates for depression and anxiety often exceeding 20% in many countries [18].

The data also revealed that psychosocial factors play a critical role in adolescent mental health. Among these factors, high levels of study pressure, loneliness, poor social relationships, and self-abusive behavior emerged as significant risk factors for mental illness. As the severity of these factors increases, so do the odds of psychological distress. Existing research has shown that loneliness is especially common among children and adolescents, female students, and those with poor economic status [19]. Further, these findings significantly address its association with depression, anxiety, and stress, which was consistent with existing literature [20]. These results underscore the multifaceted nature of adolescent mental health and highlight the need to address not only individual but also communal and relational contributors to mental well-being.

The patterns observed may be further explained by salient cultural and contextual moderators. Within collectivistic societies like China, harmonious social integration is highly valued; thus, perceived failures in social relationships (loneliness, peer difficulties) may carry an amplified psychological weight, potentially exacerbating their association with internalizing symptoms. Additionally, persistent stigma surrounding mental illness may lead to underreporting of distress in self-report measures while increasing the psychological burden for those affected, potentially intensifying the link between risk factors and outcomes. The deeply ingrained academic norms and high-stakes examination culture (Gaokao) likely magnify the perceived impact of study pressure, making it a uniquely potent contextual stressor in the Chinese educational landscape.

In China, the adolescent mental health crisis has garnered increasing policy attention, particularly in relation to the intense competition within the educational system. In response, the government launched the “Double Reduction” policy in 2021, aimed at reducing excessive homework and off-campus tutoring in compulsory education [21]. This policy was introduced not only to address educational inequality but also to alleviate the academic burden contributing to students’ distress [22]. Significant regional differences were observed in China. For instance, previous studies revealed significant regional disparities in depressive and anxious symptoms among Chinese adolescents, with higher levels observed in Western China [23]. These figures highlight a concerning national pattern while underscoring the need for region-specific data to account for local socio-economic and educational contexts.

The study’s findings also raise important implications for educational and public health policies. Despite the recent launch of China’s “Double Reduction” policy intended to alleviate academic burden, the high prevalence of study-related stress suggests that deeper structural changes in mental health education may be needed [24]. Mental health promotion must be integrated into the fabric of educational institutions, with increased availability of school-based mental health services, training for teachers in psychological literacy, and efforts to create supportive peer and family environments [25]. Basically, school-based preventive interventions should move beyond a sole focus on academic counseling to implement multi-tiered systems of support.

A key strength of this study lies in its large, diverse sample size and the use of validated measurement tools (DASS-21 and MHT), which provide reliable assessments of both mental health symptoms and associated risk factors. However, several limitations should be acknowledged. First, the cross-sectional study design precludes any causal inferences about the relationship between risk factors and mental health outcomes. Second, the reliance on self-reported data may introduce response biases, particularly underreporting due to stigma. Third, although the sample included students from primary, middle, and high schools, regional differences and school-specific stressors were not examined, which may limit generalizability. Furthermore, although the MHT study anxiety and DASS-21 anxiety are theoretically and empirically distinct, potential conceptual bias remains.

## Conclusions

This study provides large-scale, quantitative evidence of the prevalence and severity of mental health challenges among primary and secondary school students in South China, identifying academic pressure, loneliness, social relationship difficulties, and self-harm as key associated risk factors. These findings suggest the potential value of comprehensive, multi-level interventions that address academic pressure, enhance social support systems, and promote early identification and care for at-risk youth. The findings also point to the possible benefit of teacher training in adolescent mental health literacy to improve recognition of distress and facilitate timely referral, and establish structured peer-support systems and incorporating student well-being into the curriculum to build resilience and foster connectedness.

School-based mental health education, early detection, and family-level support systems may warrant further strengthening to address the complex psychosocial needs of adolescents. Future policies and programs could consider integrating mental health promotion into the broader educational and social development framework to foster a healthier, more resilient youth population in South China. Future research should employ longitudinal designs to clarify temporal pathways and conduct intervention-based studies to rigorously evaluate the effectiveness of the recommended school-based programs.

## Data Availability

The data generated and analyzed during this study are not publicly available due to the sensitive nature of mental health data and the ethical restrictions imposed by the Research Ethics Committee of Jiaxing University. The data that support the findings of this study are available from the first author (FW) upon reasonable request and with the permission of the ethics committee.
